# A Chapter about the Books

**Published:** 1867-08

**Authors:** 


### *A Chapter about the Books.*

AITKEN'S PRACTICE.—This is a magnificent work, valuable on account of the extent of its compilations and the amount of its original matter. The additions made to the American edition by Dr. Clymer, are large and valuable. It is not sufficient to say that it should be in every physician's library. It should be read *con amore* until its contents are thoroughly received and digested. The editor has taken great pleasure in recommending it as a text-book for his department in Rush Med. College.

OBSTETRICS; the Science and Art. By Charles D. Meigs, M.D., &c.—*Clarum et venerabile nomen!* The fifth and revised edition of this delightfully written and printed work is before us. It is another evidence that the American people have learned to read American books. We do not propose to "carry coals to Newcastle" in again recommending it to our readers.

PRACTICAL DISSECTIONS. By Hodge's (*vid.* p. 226 Journal), Second and revised edition. The best practical guide to the student of anatomy yet published.

INHALATIONS: by Da Costa. Full and complete instructions and suggestions by the well known and accomplished author, in the use of anatomized fluids etc., in Respiratory Diseases. An excellent monograph.

THE INDIGESTIONS by THOS. K. CHAMBERS. Whatever Dr. Chambers writes is worth buying and reading. Of course, this is a good book. No practitioner of the present time has any right to omit perusal of Dr. Chambers' writings. The JOURNAL wishes he would write a systematic work on *Practice.* The present work is clear, practical, and satisfactory. We place it among leading works of study and reference.

WHY NOT? A book for every woman, prize essay etc., H. R. STORER. A book which should be put into the hands of every woman who applies to a physician to have an abortion procured. A book which should be put into the hands of no other woman. A book worthy of being read and "whose failings lean towards virtue's side." A book which amid an ocean of twaddle on the subject, really has some value. A book which we should no more think of recommending for indiscriminate perusal by women, than to put treatises on preternatural labors into a nunnery.

REPORT ON EPIDEMIC CHOLERA in the Army of the United States in the year 1866. "Circular No. 5."

Our thanks are due to Surgeon-General Barnes for this valuable contributiion to the literature of Cholera. The report is submitted to the Department by the indefatigable, and always reliable, Brevet Lt. Col. J. J. Woodward, Assistant Surgeon United States Army.

The subordinate report by A. A. Surgeon B. F. Craig, on Disinfecting Agents is worthy of especial mention. We should reproduce it in full did space permit. As Prof. Blaney has promised us a paper on this subject at an early date and possibly for the present number of the JOURNAL, we the less regret this inability.

We are happy in the belief that by no class of officials in the world is medical science, as a whole, being more decidedly advanced than by the Medical Staff of the U. S. Army. It is a luxury to peruse the broad quarto pages on which their communications find publicity, and greater still to feel meanwhile that the mind is being fed with solid food.

WATSON ABRIDGED, (JOURNAL p. 227). An abridgement we thoroughly approve. Let students buy this: Flint on Practice, Aitken likewise, and then sandwich in Chambers', Bennett and a few of the best treatises extant on Physiology, and they will not go far astray in practice, provided, they have learned how to think on medical subjects, which, after all, is the real object of the course of medical pupillage.

ESSENTIALS OF THE PRINCIPLES AND PRACTICE OF MEDICINE. A handy-book for students and practitioners. By Henry Hartshorne, M.D., etc. Philadelphia: Henry C. Lea. 1867.

NOTES ON THE ORIGIN, NATURE, PREVENTION AND TREATMENT OF ASIATIC CHOLERA. By JOHN C. PETERS, M.D. Second edition with an appendix. New York: D. Van Nostrand, 192 Broadway. 1867. pp. 200. This work was noticed at length in the Oct. number of last year. We are pleased to find that it has been so well appreciated by the profession. For sale by W. B. Keen & Co.

ERICHSEN ON NERVOUS INJURIES. HENRY C. LEA, Philadelphia. 1867. pp. 103.

The latter of these books refers principally to railway injuries, the former to those of every variety. Aside from their interest to the general practitioner, they will be read with peculiar interest by the medical witness. Malingering in these cases has been reduced to a system in these days. It is safe to say that of a hundred persons on a railway at the time of a collision, one at least will claim damages for injury of the spine. The thing has grown to be one of the *opprobria medicorum.* Recoveries are unprofessionally rapid after a favorable verdict. Nevertheless the fancied obscurity of the subject is vastly cleared up, by a modicum of common sense applied to the facts. Let each for himself read, ponder, and understand.

PHYSIOLOGY OF THE HEART. A Lecture By CLAUDE BERNARD. Translated By J. S. MOREL, M.D., Savannah, Georgia. Purse & Son, Savannah. 1867. pp. 47. From the Translator.

Our thanks are due to Dr. Morel for this very interesting and valuable pamphlet. Its typography and illustrations do credit to the publishers, and the translator has performed his part of the work excellently. The tracings of the pulsations of the heart by the Cardiagraphic, or Sphygmographic apparatus of Mr. Marey, are well designed. The tracings are given of pulsations of the rabbit, frog, tortoise, etc. The development of the heart is closely studied, and its innervation mapped out. The lecture is invaluable to those who seek light in this important physiological topic.

INJURIES OF THE SPINE; with an Analysis of nearly four hundred cases. ASHURST, (JOURNAL p. 227). J. B. LIPPINCOTT & Co.

